# Adult systemic cat scratch disease associated with therapy for hepatitis C

**DOI:** 10.1186/1471-2334-7-8

**Published:** 2007-02-23

**Authors:** Zahida Bhatti, Charles S Berenson

**Affiliations:** 1 Infectious Disease Division, VA Western New York Healthcare System, State University of New York at Buffalo School of Medicine, Buffalo, New York 14215, USA

## Abstract

**Background:**

We describe the first case of systemic cat scratch disease in a patient receiving peginterferon α-2a and ribavirin for treatment of hepatitis C. Cases of adult systemic CSD are extremely infrequent and immunomodulatory treatment for hepatitis C has been associated with aberrant host responses to common pathogens.

**Case presentation:**

A 52 year old man being treated for hepatitis C presented with diffuse lymphadenopathy, weight loss, fevers and splenic lesions. Symptoms were initially confused with adverse effects of his regimen, delaying recognition of his infection. Diagnostic investigation, including histopathology, microbiology and serologic parameters, confirmed that his illness was due to disseminated cat scratch disease with *Bartonella henselae*.

**Conclusion:**

Disseminated CSD is exceptionally rare in adults. We describe the first case of disseminated cat scratch disease associated with peginterferon α and ribavirin to alert clinicians of the need to be aware of unusual manifestations of common infections in this population.

## Background

Cat scratch disease (CSD) most commonly presents as a localized granulomatous and suppurative lymphadenopathy caused by *Bartonella henselae*, a small fastidious gram-negative argyrophillic bacillus [1,2]. While the overwhelming majority of systemic CSD occurs in children, especially with hepatosplenic involvement, disseminated CSD is exceedingly rare in immunocompetent adults [3,4].

Interferon α and ribavirin are mainstays of treatment for chronic hepatitis C, that have been associated with aberrant immune-mediated host responses, that may resemble adverse pharmacologic effects. In this report, we present the first description of disseminated CSD in an adult receiving interferon α˜
 MathType@MTEF@5@5@+=feaafiart1ev1aaatCvAUfKttLearuWrP9MDH5MBPbIqV92AaeXatLxBI9gBaebbnrfifHhDYfgasaacH8akY=wiFfYdH8Gipec8Eeeu0xXdbba9frFj0=OqFfea0dXdd9vqai=hGuQ8kuc9pgc9s8qqaq=dirpe0xb9q8qiLsFr0=vr0=vr0dc8meaabaqaciaacaGaaeqabaqabeGadaaakeaacuaHXoqygaacaaaa@2E5A@2a and ribavirin for treatment of chronic hepatitis C, in whom signs and symptoms were initially confused with adverse effects of medications. We offer our experience to alert clinicians of the need for awareness of unusual manifestations of commons infections in this population.

## Case Presentation

A 52-year-old male with a history of hepatitis C, genotype 1b, was nearing completion of a one-year course of treatment with pegylated interferon α˜
 MathType@MTEF@5@5@+=feaafiart1ev1aaatCvAUfKttLearuWrP9MDH5MBPbIqV92AaeXatLxBI9gBaebbnrfifHhDYfgasaacH8akY=wiFfYdH8Gipec8Eeeu0xXdbba9frFj0=OqFfea0dXdd9vqai=hGuQ8kuc9pgc9s8qqaq=dirpe0xb9q8qiLsFr0=vr0=vr0dc8meaabaqaciaacaGaaeqabaqabeGadaaakeaacuaHXoqygaacaaaa@2E5A@2a (180 ug subcutaneously each week) and ribavirin (1200 mg orally each day). His initial hepatitis C viral RNA had declined from 2.2 × 10^6 ^IU/ml at onset of treatment, to <65 IU/ml by six months, and remained undetectable thereafter. Toward the end of his course of treatment, he developed fatigue, malaise, drenching night sweats, intermittent fever and chills. On the last visit for hepatitis C treatment, axillary and cervical lymphadenopathy was noticed. His symptoms were initially attributed to adverse effects of interferon α, prompting premature discontinuation of treatment after 10.5 months. One month after onset of symptoms, he presented to a nearby hospital for further workup.

He was transferred to the Buffalo VA Western New York Healthcare System with complaints of malaise and left sided mid back pain. He had a documented weight loss of 40 lbs over the previous year. He appeared chronically ill and fatigued. He had a temperature of 101°F and a heart rate of 105 beats per minute. Generalized lymphadenopathy was noted, including cervical, axillary, inguinal and right epitrochlear lymph nodes. The nodes were 1–2 cm wide, firm, movable and nontender. He had left sided abdominal fullness and mild tenderness, but no guarding or rebound. The remainder of his examination was noncontributory.

CBC revealed hemoglobin of 11.7 g/dl (13.5–17), white blood cell count of 7.6 K/cmm (4.4–10.7), platelet count of 511 K/cmm (140–375) and ESR of 80 mm/hr. Serum chemistries included a sodium of 130 mEq/L (135–145), creatinine of 0.9 mg/dl (0.7–1.4). SGOT was 68 units/l (12–34), SGPT: 95 units/l (25–65). A serum ELISA for human immunodeficiency virus was negative. Rheumatoid and anti-nuclear antibody titers were also negative.

**Figure 1:**
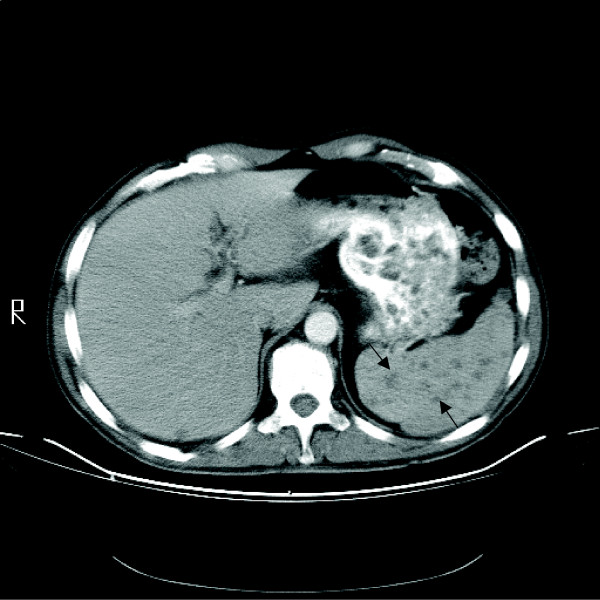
Cross sectional cut of CT scan of abdomen at the time of clinical presentation. Multiple hypoechoic densities are present in the spleen (arrows).

Computerized tomography (CT) scan of chest and abdomen revealed mild lymphadenopathy and multiple contrast enhancing hypodense lesions in the spleen (Figure 1).

Initial clinical suspicion included lymphoma and he underwent a lymph node biopsy. Further history revealed exposure to numerous cats. He frequented a neighbor who had 6–9 cats, including kittens, with which the patient had played, and from whom he received numerous scratches. Serologic studies were sent for antibodies to Bartonella, Chlamydia, Toxoplasma and Brucella. He was given ibuprofen for symptomatic relief.

**Figure 2:**
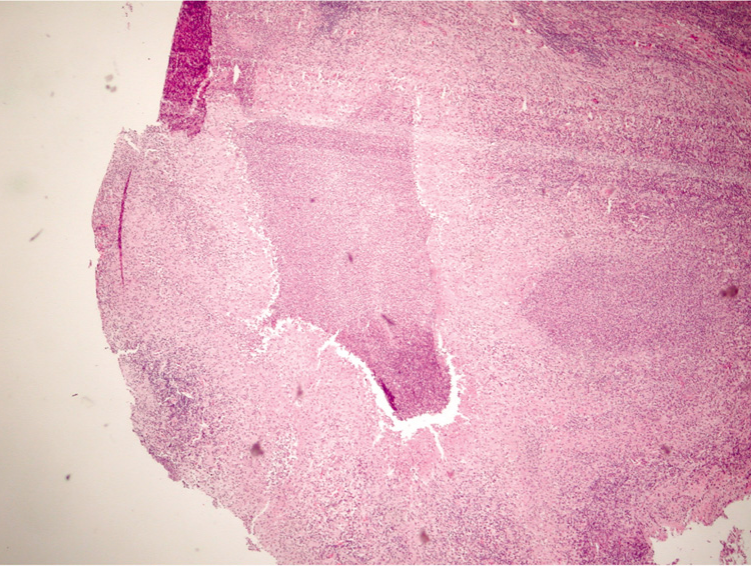
Hematoxylin and eosin stain of lymph node biopsy showing a stellate granuloma and microabscesses.

A biopsy of an epitrochlear lymph node displayed necrotizing granulomata with peripheral palisading epithelioid cells, with an admixture of plasma cells and lymphocytes. Areas of stellate necrosis with microabscesses were evident, consistent with cat scratch disease (Figure 2). Stains for acid-fast bacilli and fungi showed no organisms. Lymph node biopsy material was cultured for routine pathogens, acid-fast bacilli and fungal organisms. Although the Gram stain displayed abundant white blood cells, all cultures were sterile.

Over the next 3–4 days, the patient defervesced and improved symptomatically on ibuprofen alone. By the eighth day, serum antibody titers (IgG and IgM) for *Bartonella henselae *were reported as >1:16,384. He continued to improve without further treatment. Six months later he was doing well and had regained 25 lbs of weight. An abdominal CT scan revealed complete resolution of splenic lesions.

## Conclusion

This is the first reported case of systemic CSD with generalized lymphadenopathy and splenic involvement in an adult associated with immunomodulatory treatment for hepatitis C. In our patient, the diagnosis of CSD was established by: 1) history of exposure to cats, 2) obtaining sterile pus from a lymph node with no growth on routine cultures, 3) lymph node biopsy displaying classic findings of necrotizing stellate granulomata with microabscesses, and 4) an extremely high titer serum antibody response to *B. henselae*.

Cat scratch disease was first described in 1950 [5], although manifestations that constitute the disease have been known for over 100 years. Typically lesions may appear at the site of a cat scratch, often from a young kitten, as a papule, pustule or vesicle, followed by fever, regional lymphadenopathy and occasional systemic symptoms. Illness is usually self-limited, resolving in 2–3 months with no treatment. Atypical signs and symptoms occur most often in children and include Parinaud's oculoglandular syndrome (conjunctivitis, conjunctival granulomata with preauricular lymphadenopathy), encephalitis, myelitis, hepatosplenic disease and dissemination [6,7]. Rarely, splenic CSD may require splenectomy [8,9]. The clinical resemblance to lymphoma, of splenic abscesses in disseminated CSD such as our patient presented with, has been described [10].

Laboratory diagnosis of CSD includes appropriate histological findings and detection of serum antibodies to *B. henselae *by enzyme immunoassay, which has a specificity of up to 95% and sensitivity of 83–95%, when IgM titer is greater than 1:250 [11,12]. PCR assay of tissue or blood, although not employed routinely, may have high sensitivity and specificity [13]. PCR techniques have been applied to immunofluorescent detection of *B. henselae *in tissue sections. Although sensitivity of this method has been variable, high specificity of positive samples may be of value when the diagnosis is in question [14,15]. Recent reports of high sensitivity of immunohistochemistry with monoclonal antibodies to *B. henselae*, tested on a limited numbers of tissue samples, suggest potential future value [16]. Although well characterized, these techniques are not universally employed [17].

While the specific factors that permit dissemination of CSD are not known, clinical experience confirms that the host response to *B. henselae *contributes to the manifestation of disease. This is illustrated by the experience with *B. henselae *in AIDS patients, where the same pathogen that causes self-limited regional lymphadenopathy in immunocompetent hosts, causes bacillary angiomatosis and peliosis hepatis [18]. However, other immunodeficient states, such as chronic lymphocytic leukemia and T cell lymphoma, can also trigger dissemination of CSD [19,20]. In fact, the immunomodulatory effects of co-infection with EBV may have been responsible for one case of disseminated CSD [21].

Although the impact of interferon α and ribavirin on host immune response has had limited characterization, aberrant host responses associated with this regimen are well known, including immune-mediated Graves' disease and sarcoidosis [22]. Although advanced forms of infection, including visceral leishmaniasis [23] have been reported with interferon-α and ribavirin therapy, a major focus of infectious complications has been associated with drug induced neutropenia [24]. This was clearly not the case in our patient. Interferon α exerts biological activities by binding to cell membranes receptors, initiating numerous cellular events, including upregulation of Th1 cells and modulation of immunological activities of macrophages and lymphocytes [25]. While the precise clinical relevance of these events is not known, it is difficult to ignore the temporal relationship between administration of this regimen and onset of disease in our patient, most likely due to immunomodulatory factors, aside from neutropenia, affected by this regimen. In addition, adverse effects of therapy, including flu-like symptoms, fatigue (47–64%), fever (39–46%), and rigors (35%), were also present at the onset of our patient's illness, resulting in delayed recognition of his infection [26].

The success and increasing utilization of interferon α and ribavirin for treatment of hepatitis C will undoubtedly reveal a greater number of interferon-related side effects in the future. We offer the experience of our patient to alert clinicians of the need to be aware of aberrant presentations of infections in this population.

## Competing interests

The author(s) declare that they have no competing interests.

## Authors' contributions

Both authors (ZB and CSB) contributed equally to the research and design of this manuscript.

## Pre-publication history

The pre-publication history for this paper can be accessed here:


